# Unusual Presentation of T-cell Large Granular Lymphocytic Leukemia

**DOI:** 10.7759/cureus.26742

**Published:** 2022-07-11

**Authors:** Fawzi Abu Rous, Radhika Gutta, Rebecca Chacko, Philip Kuriakose, Vrushali Dabak

**Affiliations:** 1 Hematology and Oncology, Henry Ford Health System, Detroit, USA; 2 Internal Medicine, Henry Ford Health System, Detroit, USA; 3 Hematology and Medical Oncology, Henry Ford Health System, Detroit, USA

**Keywords:** liver dysfunction, large granulocytic lymphocyte, liver disease, t-lgl, leukemia

## Abstract

Large granular lymphocytic (LGL) leukemia is a rare chronic lymphoproliferative disorder that can arise from T- or natural killer-cell lineages. It is an indolent disease that typically occurs in the sixth decade of life. Most cases of T-cell LGL leukemia (T-LGL) are associated with autoimmune disorders. Patients with T-LGL are generally asymptomatic; however, they can present with symptoms related to neutropenia, infections, and autoimmune disorders. Here, we report two cases of T-LGL in which the patients presented with liver dysfunction.

## Introduction

Large granular lymphocytic (LGL) leukemia is a rare clonal disorder accounting for only 2-5% of chronic lymphoproliferative disorders in Europe and North America [1]. LGL leukemia can arise from T- or natural killer-cell lineages [2]. This is an indolent disease that usually presents with neutropenia, infection, and autoimmune disorders [2]. In 1999, The World Health Organization classified LGL leukemia as a subgroup of mature peripheral T-cell neoplasms [3]. LGL leukemia differs from T-cell lymphoblastic leukemia not only in the cell of origin but also in molecular characteristics, presentation, and age of onset [4]. Here, we report two cases of T-cell LGL leukemia (T-LGL) in which the patients presented with liver dysfunction.

## Case presentation

Case one

A 48-year-old woman presented with abdominal pain and distension and 25 lb unintentional weight loss over the last two months. She denied systemic symptoms such as nausea, vomiting, diarrhea, night sweats, or fever. She had no significant medical history and did not have any history of substance abuse. Physical examination was significant for diffuse abdominal tenderness and hepatosplenomegaly. Her laboratory results are shown in Table 1.

**Table 1:** Case one: laboratory results.

Lab test	Result	Reference
White blood cell count	9.7 × 10^9^/L	3.8–10.6 × 10^9^/L
Absolute lymphocyte count	6.01 × 10^9^/L (high)	1.10–4.00 × 10^9^/L
Absolute neutrophil count	1.94 × 10^9^/L	1.80–7.70 × 10^9^/L
Hemoglobin	135 g/L	120–150 g/L
Platelets	223 × 10^9^/L	150–450 × 10^9^/L
Alanine aminotransferase	16 IU/L	<52 IU/L
Aspartate aminotransferase	44 IU/L (high)	<35 IU/L
Alkaline phosphatase	299 IU/L (high)	40–140 IU/L
Hepatitis B and C serologies	Negative	
Ferritin	43 μg/L	11.0–307.0 μg/L
Ceruloplasmin	410 μmol/L	200–600 μmol/L
α-1 antitrypsin	2.27 g/L	0.9–2.00 g/L
Autoimmune hepatitis panel	Negative	
Antinuclear antibodies	Negative	
Rheumatoid factor	<10 IU/mL	<14 IU/mL

**Figure 1:**
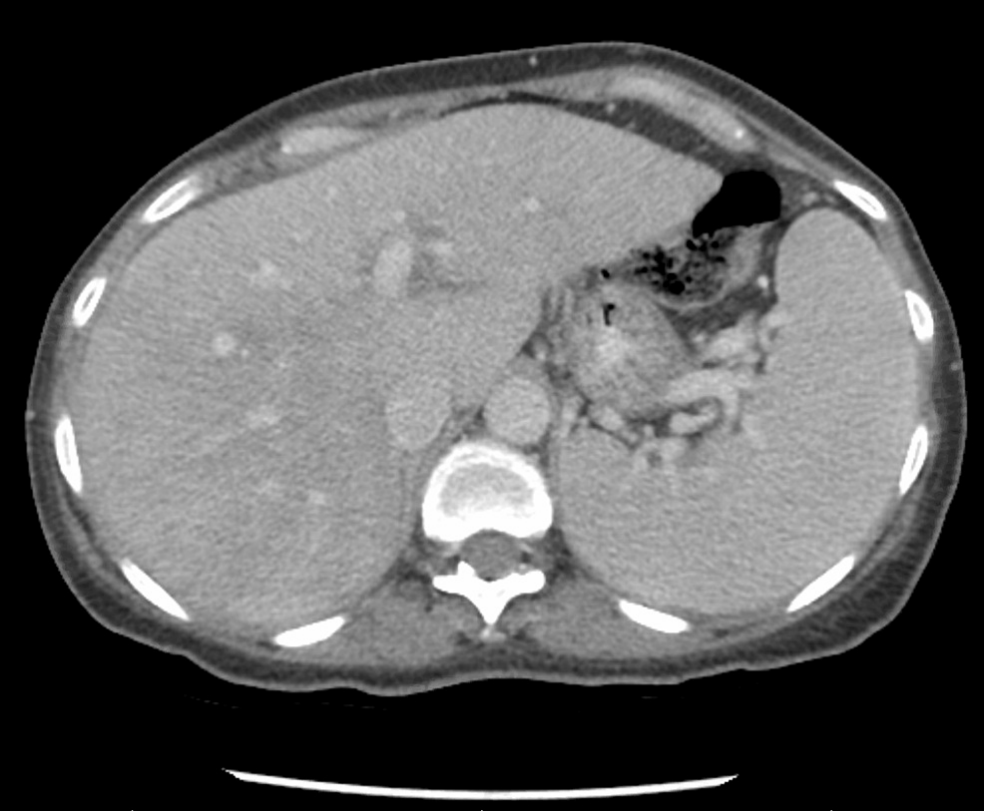
Case one: computed tomography of the abdomen and pelvis. Findings compatible with cirrhotic liver with secondary features of portal hypertension such as splenomegaly, dilated portal and splenic veins, and minimal ascites

**Figure 2:**
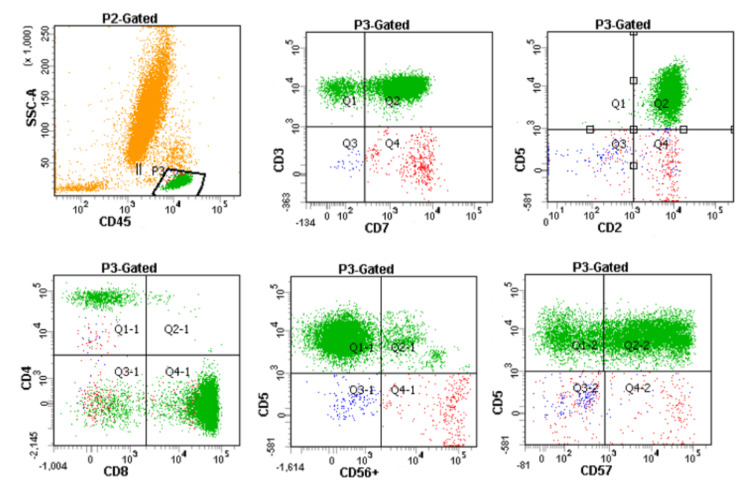
Case one: bone marrow flow cytometry. P3-gated total lymphocytes in bone marrow aspirate show CD8+, CD57+, TCR beta+ abnormal T-cells consistent with T-cell large granular lymphocytic leukemia. P3: inositol 1,4,5-triphosphate receptor; SSC-A: side scatter parameter-A; TCR: T-cell receptor

Computed tomography (CT) of the abdomen and pelvis demonstrated a cirrhotic liver with portal hypertension, small ascites, and splenomegaly (Figure 1). Given the findings on imaging and the unremarkable workup for hepatic cirrhosis, a liver biopsy was performed and showed marked infiltration by atypical lymphocytes in a multifocal nodular and intrasinusoidal distribution. Immunohistochemistry was positive for T-cell receptor (TCR) rearrangement, and flow cytometry showed an aberrant CD8+, CD57+, TCR beta+ T-lymphocyte population. Hepatosplenic T-cell lymphoma was ruled out by negative fluorescence in situ hybridization testing for isochromosome 7q. Bone marrow examination showed a similar clone of aberrant T-lymphocytes by flow cytometry (Figure 2) occupying 15% of the marrow with normal cytogenetics. Hence, the diagnosis of T-LGL leukemia was made based on the results of the liver and bone marrow biopsy. The patient was started on weekly methotrexate (10 mg/m^2^) and prednisone taper (starting with 1 mg/kg/day), with which her symptoms, liver enzymes, and lymphocyte count improved.

Case two

**Figure 3:**
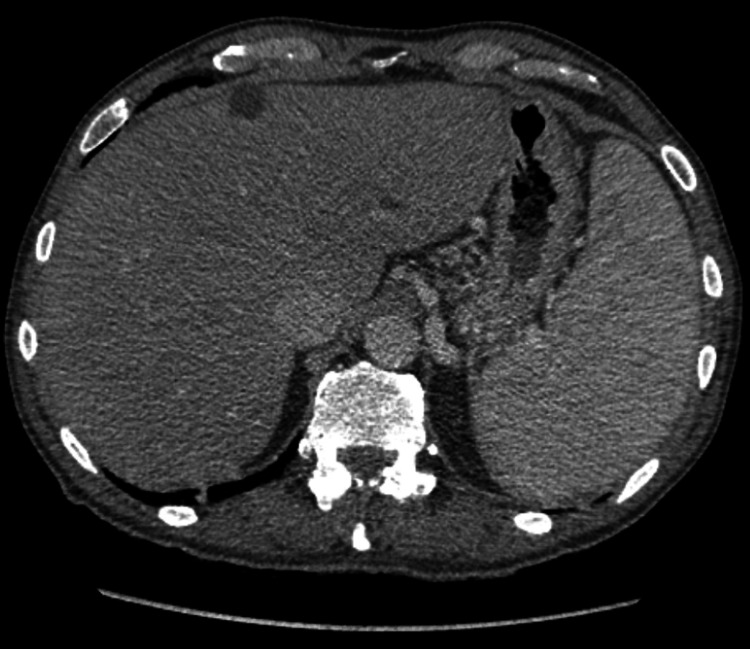
Case two: computed tomography of the abdomen and pelvis. Findings compatible with portal venous hypertension including splenomegaly and multiple collaterals.

A 75-year-old male with a medical history of psoriatic arthritis, previously treated with methotrexate and etanercept, presented with nausea, vomiting, and a 30-pound unintentional weight loss over several months. Physical examination was remarkable for splenomegaly. Initial laboratory results are provided in Table 2. CT of the abdomen and pelvis revealed portal venous hypertension, hepatosplenomegaly, and gallbladder wall thickening (Figure 3).

**Table 2:** Case two: laboratory results.

Lab test	Result	Reference
White blood cell count	1.2 × 10^9^/L (low)	3.8–10.6 × 10^9^/L
Absolute lymphocyte count	0.91 × 10^9^/L (low)	1.10–4.00 × 10^9^/L
Absolute neutrophil count	0.04 × 10^9^/L (low)	1.80–7.70 × 10^9^/L
Hemoglobin	106 g/L (low)	120–150 g/L
Platelets	47 × 10^9^/L (low)	150–450 × 10^9^/L
Alanine aminotransferase	12 IU/L	<52 IU/L
Aspartate aminotransferase	30 IU/L	<35 IU/L
Alkaline phosphatase	74 IU/L	40–140 IU/L
Hepatitis B and C serologies	Negative	
Ferritin	46 μg/L	24.0–336.0 μg/L
Autoimmune hepatitis panel	Smooth muscle antibody: 80 units	Smooth muscle antibody: <20 units
Antinuclear antibodies	1:640 (high)	<1:80
Rheumatoid factor	328 IU/mL (high)	<14 IU/mL

**Figure 4:**
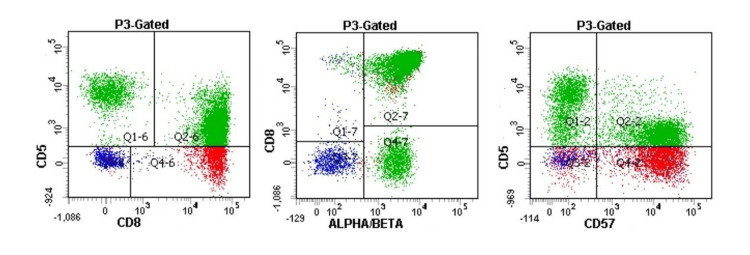
Case two: bone marrow flow cytometry. P3-gated total lymphocytes in bone marrow aspirate show CD8+, CD5 dim to negative, CD57+, TCR alpha/beta+ abnormal T-cells consistent with T-cell large granular lymphocytic leukemia. P3: inositol 1,4,5-triphosphate receptor; TCR: T-cell receptor

Bone marrow and liver biopsy were pursued. His liver biopsy showed lymphocytes infiltrating the parenchyma in a sinusoidal pattern. Immunohistochemistry was positive for CD3, CD8, and CD57. The polymerase chain reaction was positive for alpha/beta TCR. Bone marrow biopsy revealed a hypercellular marrow with atypical interstitial and intrasinusoidal lymphocytic infiltrates. Flow cytometry revealed aberrant CD8+, CD57+, and TCR alpha/beta T-cell population (Figure 4). Fluorescence in situ hybridization and cytogenetic studies were negative. The findings on the liver and bone marrow biopsies were consistent with a diagnosis of T-LGL. He was started on weekly methotrexate (10 mg/m^2^) and daily prednisone (1 mg/kg/day), tapered to his clinical status, with which his symptoms and functional status improved, and his pancytopenia remained stable without further decline.

## Discussion

LGL leukemia is an uncommon lymphoproliferative disorder that can be of T- or natural killer-cell lineage affecting one in every 10 million people in the United States [1]. Patients with T-LGL typically present in their sixth decade of life with neutropenia and recurrent bacterial infections or autoimmune disorders [2]. One-third of the patients are asymptomatic on presentation; however, others can present with generalized symptoms such as weight loss, fatigue, night sweats, and fevers [5]. T-LGL leukemia is most commonly associated with rheumatoid arthritis (RA) in 40% of the cases; however, it can be associated with other autoimmune disorders. Additionally, other hematologic disorders such as myelodysplastic syndrome, autoimmune-related cytopenia, and Hodgkin’s lymphoma can be associated with T-LGL [2].

LGL is usually diagnosed when cytopenia is noticed on routine blood work. Although on examination lymphadenopathy is rare and hepatosplenomegaly is not usually appreciated, around two-thirds of patients have splenomegaly on imaging [6].

Patients with T-LGL usually have a normal or elevated absolute lymphocyte count with a mild elevation in the LGL count (>2,000 LGLs/µL [normal range: 200-400 LGLs/µL]) [2]. Most cases (90%) have bone marrow involvement. Nevertheless, bone marrow examination is required in patients with unusual presentations, as seen in both of our patients [2]. Immunophenotypic studies revealing a T-LGL clone that is positive for CD3, CD8, CD16, and CD57 and negative for CD4, CD56, or CD28 are needed to establish the diagnosis [6]. Detection of clonal rearrangement of TCRs is paramount in the diagnosis of T-LGL. Most cases are alpha/beta variants, while fewer than 10% are gamma/delta variants [7]. Negative isochromosome 7q on fluorescence in situ hybridization can be used to rule out hepatosplenic T-cell lymphoma [8].

There are only a few reported cases of liver involvement with T-LGL; one case series described four patients who were previously diagnosed with T-LGL and underwent liver biopsy due to abnormal liver function tests. Pathology revealed T-LGL infiltrating the hepatic sinusoids [9]. Another report described a case of aggressive T-LGL in which the patient presented with hepatosplenomegaly and subsequently developed hepatic fibrosis. Liver biopsy showed diffuse perisinusoidal fibrosis, extramedullary hematopoiesis, and lymphocytic infiltrate that was CD3 positive but CD4 and CD8 negative on immunohistochemistry, which strongly suggested they were T-LGL infiltrating the sinusoids [10]. All patients in these reports had bone marrow involvement with T-LGL.

There have been rare cases of LGL seen following kidney, liver, and hematopoietic stem cell transplantation. These cases were found to be of donor origin, likely a result of abnormal proliferation in response to a foreign antigen in a similar mechanism to how graft-versus-host disease develops [11]. Another unusual presentation of LGL is vasculitis, typically small-vessel vasculitides, with clinical presentations of purpura, arthralgia, peripheral neuritis, and renal glomerulonephritis [12]. Vasculitis as a presenting factor was predominantly described in women and had a high rate (80%) of complete response rate to treatment [12].

Treatment is reserved for symptomatic patients with severe neutropenia (absolute neutrophil count <500/µL), recurrent infections, transfusion-dependent or symptomatic anemia, or associated autoimmune disorders requiring treatment [5]. Asymptomatic patients can be closely monitored and do not require treatment. The mainstay of treatment is immunosuppressive therapy with methotrexate, cyclophosphamide, or cyclosporine depending on the symptoms and associated disorders [5]. For example, methotrexate and prednisone (1 mg/kg/day for a month followed by a taper) would be the treatment of choice in a patient with RA and T-LGL. History, physical examination, and complete blood count with differential should guide response to treatment. Complete remission is defined as a circulating LGL count of fewer than 500 LGLs/µL with normalization of blood counts. Response has been reported in around 50% of patients with time to response between two and 12 weeks and a median duration of response between two and four years [1,5]. Patients with a partial response should be maintained on their current treatment until confirmed failure or until the maximum duration for that regimen has elapsed [1,5].

## Conclusions

T-LGL is an uncommon disease that can be associated with RA and typically presents with neutropenia. It rarely presents with hepatic manifestations, as in the patients presented in this report. The diagnosis is based on the detection of aberrant CD8+, CD57+, and TCR alpha/beta+ T-LGL population. Treatment, when necessary, is immunosuppressive using agents such as methotrexate, cyclophosphamide, and cyclosporine.
